# Blue and red LEDs modulate polyphenol production in Precoce and Tardiva cultivars of *Cichorium intybus* L.

**DOI:** 10.3389/fpls.2025.1529804

**Published:** 2025-02-21

**Authors:** Felicia Menicucci, Giovanni Marino, Fabiano Sillo, Andrea Carli, Luana Beatriz dos Santos Nascimento, Cassandra Detti, Mauro Centritto, Cecilia Brunetti, Raffaella Maria Balestrini

**Affiliations:** ^1^ Institute for Sustainable Plant Protection, National Research Council, Sesto Fiorentino, Italy; ^2^ Institute for Sustainable Plant Protection, National Research Council, Turin, Italy; ^3^ Department of Agriculture, Food, Environment and Forestry (DAGRI), University of Florence, Firenze, Italy; ^4^ Centro de Ciências da Saúde, Federal University of Rio de Janeiro (UFRJ), Rio de Janeiro, Brazil; ^5^ Institute of Biosciences and Bioresources, National Research Council, Bari, Italy

**Keywords:** *Cichorium intybus* L., light-emitting diodes (LEDs), polyphenols, chlorophylls, HPLC-DAD-Q-ToF/MS

## Abstract

**Introduction:**

Plant growth and metabolism can be optimized by manipulating light intensity and wavelength. Since the use of light-emitting diodes (LEDs) allows easy regulation of the light spectrum, LEDs technology is largely tested to produce high-quality food. Red leaf chicory is a horticultural plant of high commercial value, rich in vitamins, minerals and phytochemical compounds with bioprotective and antioxidant roles.

**Methods:**

*Cichorium intybus* L. (Asteraceae family) seedlings of the cultivar Rossa di Treviso Precoce and Rossa di Treviso Tardiva were cultivated under blue and red LEDs for three to four weeks, whereas white LEDs, proving full visible light spectrum, were supplied as control. The leaf polyphenols were characterized and quantified by HPLC-DAD-Q-ToF analysis, the leaf chlorophyll content was measured using a handheld optical analyzer and the photosystem II efficiency was assessed with a porometer-fluorometer.

**Results:**

The PS II efficiency decreased in response to red LEDs treatment only. The highest levels of polyphenol and chlorophyll content were registered in response to blue LEDs exposure in both cultivars. The Rossa di Treviso Tardiva also exhibited a significant accumulation of polyphenols under red LEDs compared to white LEDs. The polyphenolic composition of the two cultivars significantly changed depending on the type of LEDs used. The leaf extracts of plants grown under red LEDs showed a prevalence of kaempferol 3-O-glucuronide, whereas a predominance of quercetin derivatives was found in response to white and blue LEDs. The comparison of the two cultivars revealed that the Rossa di Treviso Precoce was characterized by a higher content of polyphenols, independently of the type of LEDs.

**Discussion:**

Species-specific protocols are required for producing high-content nutrient vegetables. In our study, red LEDs induced a completely different leaf polyphenol composition than blue and white LEDs, pointing out that an accurate light spectrum selection is crucial for shaping plant metabolism. Blue LEDs improved the content of photosynthetic pigments and induced an accumulation of highly antioxidant polyphenols in both Rossa di Treviso Precoce and Tardiva *C. intybus* cultivars, emerging as a valuable tool for improving their nutraceutical content.

## 1 Introduction

Chicory *- Cichorium intybus* L. – is a diploid species belonging to the Asteraceae family, widespread in all Eurasia and in the northern part of Africa. There are both wild and cultivated varieties of *C. intybus* and several cultivars can be accounted (Aldahak et al., 2021). Among the different cultivars, the “Radicchio” ones are widely cultivated in northeastern Italy, where they represent economically relevant vegetable crops. The “Rossa di Treviso Tardiva” (Late Red of Treviso) and “Rossa di Treviso Precoce” (Early Red of Treviso) cultivars are local products certified PGI (Protected Geographical Indication) and PDO (Protected Designation of Origin) (Carazzone et al., 2013; Papetti et al., 2017) in Italy. In this part of the country, the Radicchio chicories constitute, in fact, a flagship ingredient of the local cuisine. The red crunchy leaves, characterized by a bitter taste, are consumed as fresh salad as well as cooked, *e.g.*, in the “risotto” with rice, radicchio and gorgonzola cheese, as grilled radicchio, etc.

Additionally, common chicory has also a long history of medical and food uses. Formerly consumed by the Ancient Egyptians for its digestive and therapeutic properties (Janda et al., 2021), chicory extracts are extensively used against gastrointestinal disorders, as well as added to beverages to obtain functional drinks (Kim et al., 2017). The composition of its tissues, rich in micronutrients (*e.g.*, vitamins) and various high-value phytochemicals (*e.g.*, inulin, tannins, chlorophyll, coumarins, flavonoids), concentrated in both root and aerial parts, mirrors the health benefits associated to the consumption of this plant species (Kiani et al., 2023). Among polyphenols, chicoric and chlorogenic acids are highly represented in *C. intybus* (Papetti et al., 2017), and particularly, the red cultivars display high levels of anthocyanins (D’evoli et al., 2013), already known to play a key role in the prevention of cardiovascular diseases (Wallace, 2011), obesity and diabetes (Tsuda, 2012; Iqbal et al., 2021).

The production of fresh vegetables with a high content of phytonutrients is one of the main goals of the horticulture industry (Martínez-Ispizua et al., 2022). Providing and manipulating artificial light by Light-Emitting Diodes (LEDs) is one of the possible options to drive indoor cultivation in this direction (Appolloni et al., 2022). These lamps enable an easy modulation of the emitted light spectrum, which ranges from the ultraviolet to the infrared region, allowing the improvement of specific traits of interest, such as the color or the accumulation of specific compounds (Carvalho and Folta, 2014; Gómez and Izzo, 2018). For these reasons, in the last decade, LEDs lighting has emerged as dominant innovative technology, finding large use in greenhouses and indoor environments, particularly for the cultivation of leafy vegetables such as lettuce and chicory (Johkan et al., 2010; Ouzounis et al., 2015; Alrifai et al., 2019; Pennisi et al., 2019, 2020). The indoor cultivation of these microgreens through artificial crop systems such as vertical farming technology, implies additional benefits other than the production of high-nutrient-content food, the most remarkable of which include increased productivity, prevention of land consumption, water saving and reduced transportation costs (Kalantari et al., 2018; Mir et al., 2022).

For the photosynthesis, plants prefer blue (400-480 nm) and red light (600-700 nm), having chlorophyll *a* and *b* their maximum absorption in these regions (Chlorophyll *a*: 430 and 665 nm; Chlorophyll *b*: 453 and 642 nm) (Ouzounis et al., 2015; Pennisi et al., 2019). It was reported that blue light enhances the accumulation of anthocyanins and other functional compounds, whereas red light promotes leaf expansion and stem elongation in many species (Son and Oh, 2013; Carvalho and Folta, 2014; Gómez and Izzo, 2018; Pennisi et al., 2019). In this context, it is worth noting that the fraction of radiation emitted by LEDs in the blue region (420-450 nm) is much higher than that of high-pressure sodium lamps, traditionally used in greenhouses, which is approximately only 5% of the full-visible spectrum (Islam et al., 2012). Consequently, the selection of the optimal lighting turns to be pivotal for a targeted plant response and a tailored production (Gómez and Izzo, 2018; Pennisi et al., 2020).

This work aimed to assess the effect of blue and red LEDs on two *C. intybus* cultivars of high commercial value, the Rossa di Treviso Tardiva (from now called Tardiva) and the Rossa di Treviso Precoce (from now called Precoce), in comparison with full-visible spectrum light (white LEDs). Physiological parameters, such as the PS II efficiency and chlorophyll content, were measured together with leaf polyphenols content, to detect possible differences induced by the different irradiation and to determine the best cultivar and light treatment association that provide the highest amount of bioactive compounds.

## 2 Materials and methods

### 2.1 Plant cultivation and experimental set-up

Seven seedlings for two cultivars of *C. intybus*, Tardiva and Precoce (both provided by Franchi Sementi s.p.a.), were grown in 25 cc cells of polystyrene germination trays, containing a mixture of sand:peat (4:1, v:v), in a growth chamber with a temperature of 25°C and 60% of RH, for four weeks. Three groups of plants *per* cultivar were grown under three different light conditions, provided by a multi-channel LEDs lighting system (ENFIS Ltd, UK): i. White LEDs (complete visible spectrum LEDs), ii. Blue LEDs (monochromatic channel radiance, with the wavelength peak at 461 nm, set up to be 70% higher than the other light components) and iii. Red LEDs (631 wavelength peak radiance set to 70% higher than the other channels) (**Supplementary Figure S1**). In all the light treatments, the total photosynthetic photon flux density (PPFD) was 600 μmol m^–2^ s^–1^.

### 2.2 Photosystem II efficiency and chlorophyll content

After four weeks of growth under different light conditions, the photosystem II efficiency - φ_PSII_ - was measured on two leaves *per* seedling (*i.e.*, the two broadest leaves) with a handheld porometer-fluorometer (Li-600, LICOR Biosciences, USA). Measurements have been collected under white light at 600 μmol m^–2^ PPFD. Chlorophyll leaf content has been assessed at three and four weeks of growth using a handheld optical analyzer (Dualex, Force One, France).

### 2.3 Leaf extracts

Two leaves *per* plant from four plants were collected, frozen in liquid nitrogen and stored at −80°C until the moment of the extractions for biochemical analyses. Leaves from the same plants were pooled together to make individual replicates (n=4). Four replicates *per* treatment were used. Leaf fresh material (100 mg) was ground in a mortar with liquid nitrogen and then extracted with 3 × 1 mL ethanol 75% solution (pH 2.5 adjusted with formic acid) using an ultrasonic bath (BioClass^®^ CP104, Pistoia, Italy) at a constant frequency of 39 kHz and power of 100 W, during 30 min, at 5°C. After that, extracts were centrifuged (5 min, 9000 rpm, ALC^®^ 4239R, Milan, Italy), and the supernatants were partitioned with 3 × 2 mL of n-hexane to remove lipophilic compounds that could interfere with the analysis. The hydroethanolic phase was reduced to dryness using a rotavapor (BUCHI^®^ P12, Cornaredo, Italy; coupled to a vacuum controller V-855), and the residue was resuspended with 250 µL of MeOH: Milli-QH_2_O solution (1:1 v/v, pH 2.5 adjusted with formic acid). After the extraction procedure, the solution was characterized and quantified by HPLC-DAD/Q-TOF.

### 2.4 HPLC-DAD/Q-TOF analyses

The characterization and subsequent quantification of polyphenols was made by LC-QTOF (Agilent 6530C, Agilent Technologies SpA, Milan, Italy) utilizing a quadrupole mass spectrometer operating in the electrospray ionization (ESI) negative mode coupled to a diode array detector (DAD). The applied ESI parameters were as follows: capillary voltage, 4000 V; fragmentor 180 V; skimmer 60 V; OCT 1 RF Vpp 750 V; pressure of nebulizer 20 psi; drying gas temperature 325°C; sheath gas temperature 400°C. Compounds separation was performed using an Agilent Poroshell 120 Aq-C18 column (2.7 μm) applying a 40-minute linear gradient solvent passing from 97% of water acidified with 0,1% formic acid (solvent A) to 97% of acetonitrile acidified with 0,1% formic acid (solvent B). The flow rate was of 0.30 mL min^-1^ and the injection volume was 1 μL. The quantification was performed in DAD at the specific wavelengths of the different compounds using five-point calibration curves of the following standards (all from Extrasynthese, Lyon, France): caftaric acid, chlorogenic acid, chicoric acid, quercetin 7-O-glucoside, apigenin 7-O-glucoside, kaempferol 7-O-glucoside, isorhamnetin 7-O-rhamnoside, luteolin 7-O-glucoside, ferulic acid, epigallocatechin, cyanidin 3-O-glucoside chloride and cyanidin 3,5-diglucoside chloride.

### 2.5 Statistical analysis

The data were analyzed for normality of distribution (Shapiro-Wilk test) and homoscedasticity (Levine test) and when at least one of these two assumptions was missing, the non-parametric Kruskal-Wallis test for multiple comparisons, followed by the Mann-Whitney U test for pairwise comparison, was performed. For normally distributed data, a one-way ANOVA, followed by Tukey’s multiple comparison test, was performed within the same sampling time (*i.e.*, 3 or 4 weeks). For the comparison between the same treatment supplied to the two cultivars, an unpaired Student’s t-test was run. Differences were considered statistically significant at p <0.05.

## 3 Results

### 3.1 Effect of the light treatments on the photosystem II efficiency and chlorophyll content

**Figure 1:**
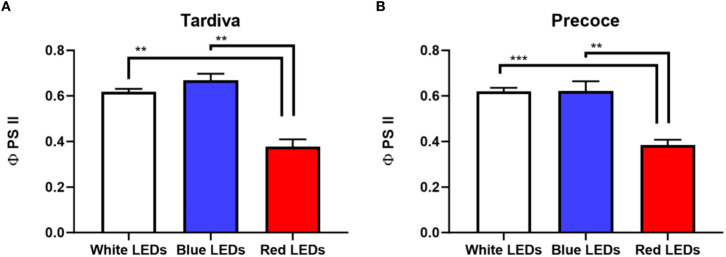
Photosystem II efficiency (φ PS2) of *C*. *intybus* Tardiva **(A)** and Precoce **(B)** cultivars after four weeks of irradiance with white, blue and red LEDs. Values are expressed as mean ± SE of 6-7 replicates per thesis (Tardiva: white LEDs n=6, blue LEDs n=7, red LEDs n=6; Precoce: white LEDs n=7, blue LEDs n=6, red LEDs n=7). Statistical differences among treatments are indicated by asterisks (** p<0.01; *** p<0.001).

The values of the photosystem II efficiency (φ PS II) measured for plants grown under red LEDs were significantly lower than those of blue and white LEDs treatments. This result was registered in both cultivars. Regarding the comparison of the two cultivars, no significant differences were observed within each single treatment (e.g. white/blue/red LEDs) (**Figures 1A, B**).

**Figure 2:**
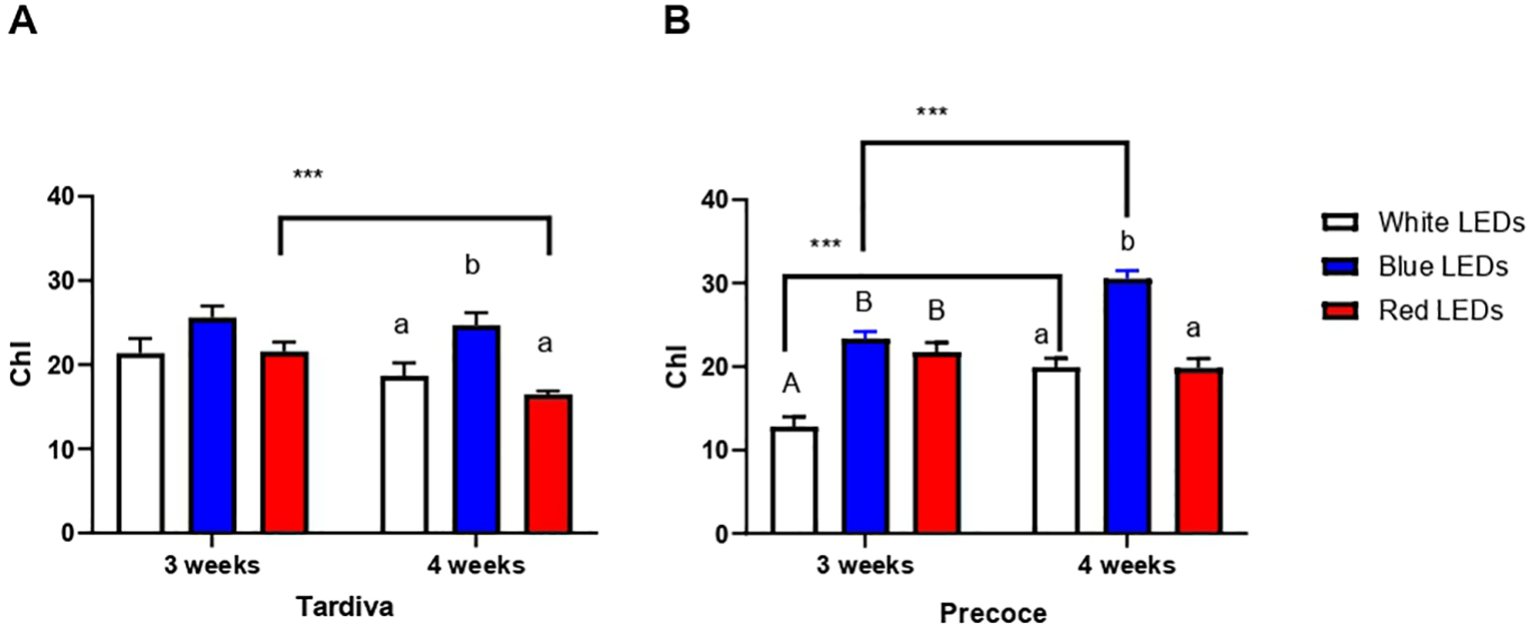
Chlorophyll content (Chl) of *C. intybus* Tardiva **(A)** and Precoce **(B)** cultivars after three and four weeks of irradiance with white, blue and red LEDs. Values are expressed as mean ± SE of 4-7 replicates *per* thesis (Tardiva 3 weeks: white LEDs n=4, blue LEDs n=7, red LEDs n=6, Tardiva 4 weeks: white LEDs n=5, blue LEDs n=6, red LEDs n=7; Precoce 3 weeks: white LEDs n=6, blue LEDs n=7, red LEDs n=7, Precoce 4 weeks: white LEDs n=7, blue LEDs n=6, red LEDs n=7). Capital letters and lowercase letters indicate significant differences among treatments after three and four weeks of irradiance, respectively. Statistical differences within the same treatment at three and four weeks are indicated by asterisks (*** p<0.001).

The chlorophyll content measured in Tardiva after three weeks of irradiance with white, blue and red LEDs did not change depending on the different light, as no significant differences were detected among the treatments (**Figure 2A**). A significant increase was registered for plants exposed to blue LEDs for four weeks, with this value being significantly higher than those observed for white and red LEDs treatments. The overtime comparison of each single treatment revealed that a significantly lower chlorophyll content was registered after four weeks of irradiance with red LEDs, whereas no significant time-dependent differences were observed for white and blue LEDs treatments (**Figure 2A**).

Considering the Precoce, significantly higher chlorophyll contents were measured after three weeks of irradiance with both blue and red LEDs with respect to white LEDs, the blue and red values being comparable to one another (**Figure 2B**). After four weeks of irradiance, plants exposed to white and red LEDs showed similar chlorophyll contents, whereas a significantly higher value was observed for blue LEDs treatment. Considering the single treatment, white and blue LEDs induced a significant increase in chlorophyll content over time, as values after four weeks were significantly higher than those registered after three weeks, whereas no time-dependent changes were observed for red treatment (**Figure 2B**).

Comparing the two cultivars, a significantly higher content of chlorophyll was registered for Precoce exposed to blue and red LEDs for four weeks, whereas the Tardiva showed a significantly higher value after three weeks of white LEDs irradiance (**Supplementary Figure S2**).

### 3.2 Effect of the LEDs treatments on the polyphenolic content

**Figure 3:**
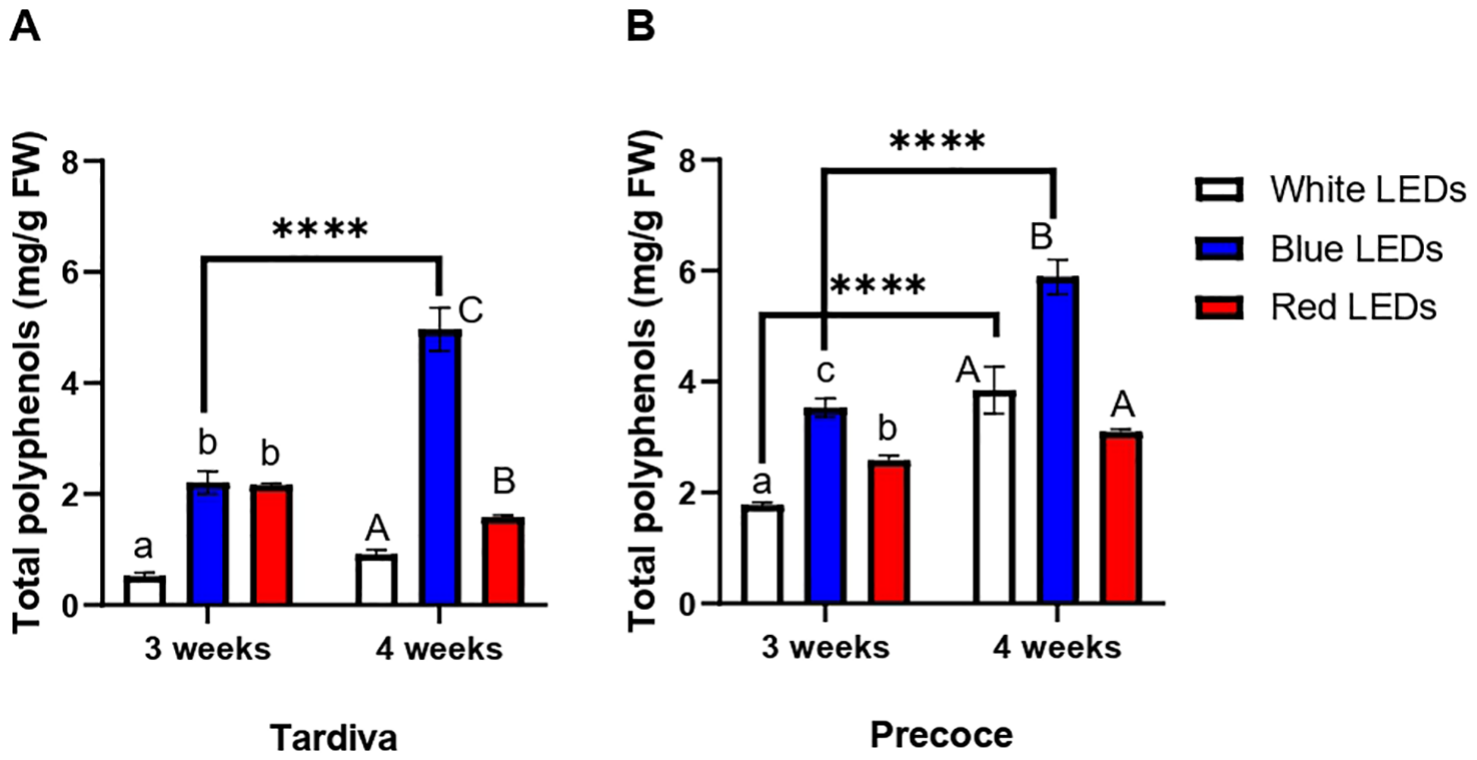
Content of polyphenols (mg/g FW) *C*. *intybus* Tardiva **(A)** and Precoce **(B)** cultivars after three and four weeks of irradiance with white, blue and red LEDs. Values are expressed as mean ± SE of 4 replicates per thesis. Capital letters and lowercase letters indicate significant differences among treatments after three and four weeks of irradiance, respectively. Statistical differences within the same treatment at three and four weeks are indicated by asterisks (**** p<0.0001).

The total content of polyphenols measured in Tardiva was significantly increased by the exposure to blue and red LEDs for three weeks, showing comparable values in the two treatments (**Figure 3A**). The highest content of polyphenols was measured in plants exposed to blue LEDs after four weeks of irradiation. In Tardiva, this value was significantly higher than in the red treatment, which in turn, significantly differed from the control (white LEDs). Blue LEDs also induced a significant increase in the total polyphenols content over time: after four weeks this content was significantly higher than that measured after three weeks (**Figure 3A**).

Looking at the Precoce, significantly higher levels of polyphenols were registered in response to blue LEDs irradiation compared to red one, after three weeks of exposure (**Figure 3B**). Red treatment also induced a significant accumulation of polyphenols compared to the control (white LEDs). After four weeks of treatment, the highest content of polyphenols was observed for blue LEDs treatment, inducing a significant accumulation of these compounds when compared to white and red LEDs. Regarding the overtime effect, a significant accumulation of polyphenols was registered in response to white and blue LEDs, whereas no significant differences were found between three and four weeks of red LEDs irradiance (**Figure 3B**).

The comparison of the two cultivars showed that Precoce has a significantly higher polyphenol content than Tardiva, regardless of the sampling time. This result was observed for all the treatments except for the blue LEDs at four weeks (**Supplementary Figure S3**).

### 3.3 Effect of the light treatments on the polyphenolic composition

**Figure 4:**
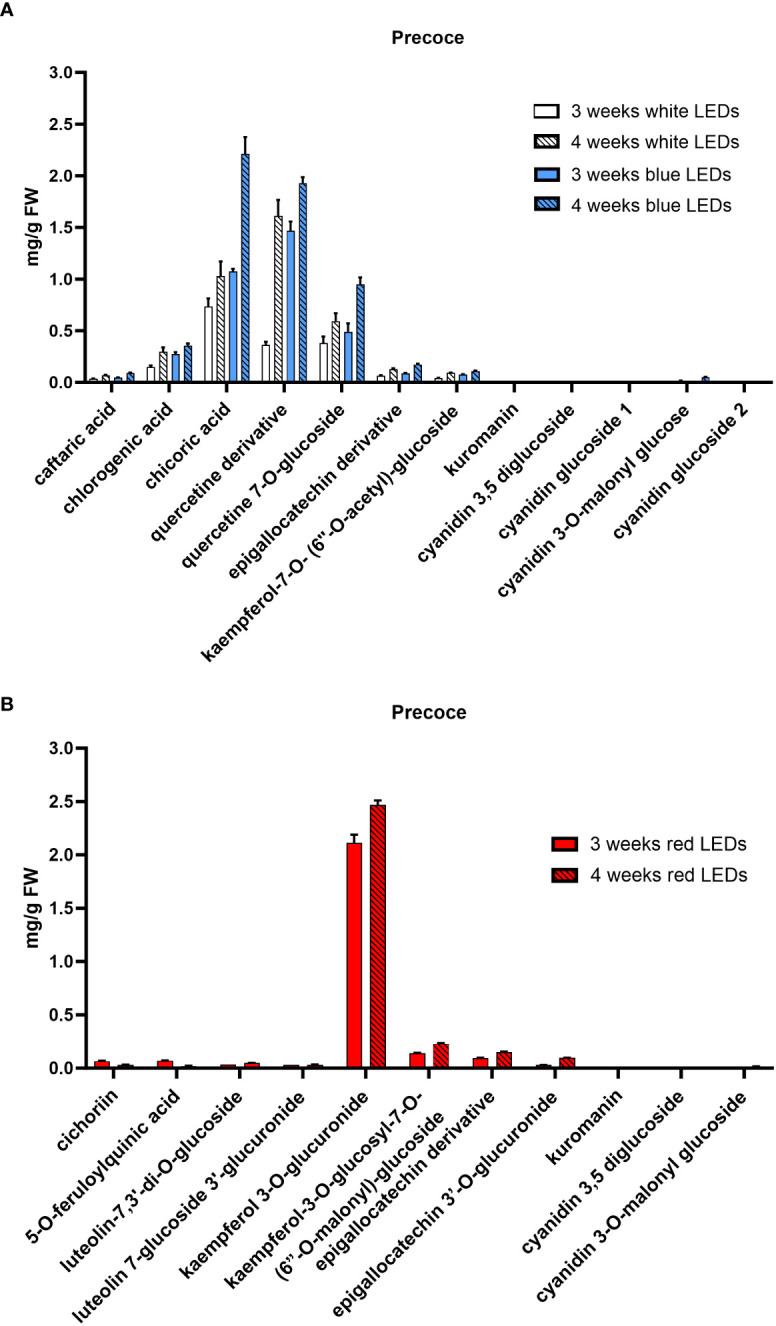
Polyphenolic composition of *C*. *intybus* Precoce cultivar after three and four weeks of treatment with white, blue **(A)** and red **(B)** LEDs.

**Figure 5:**
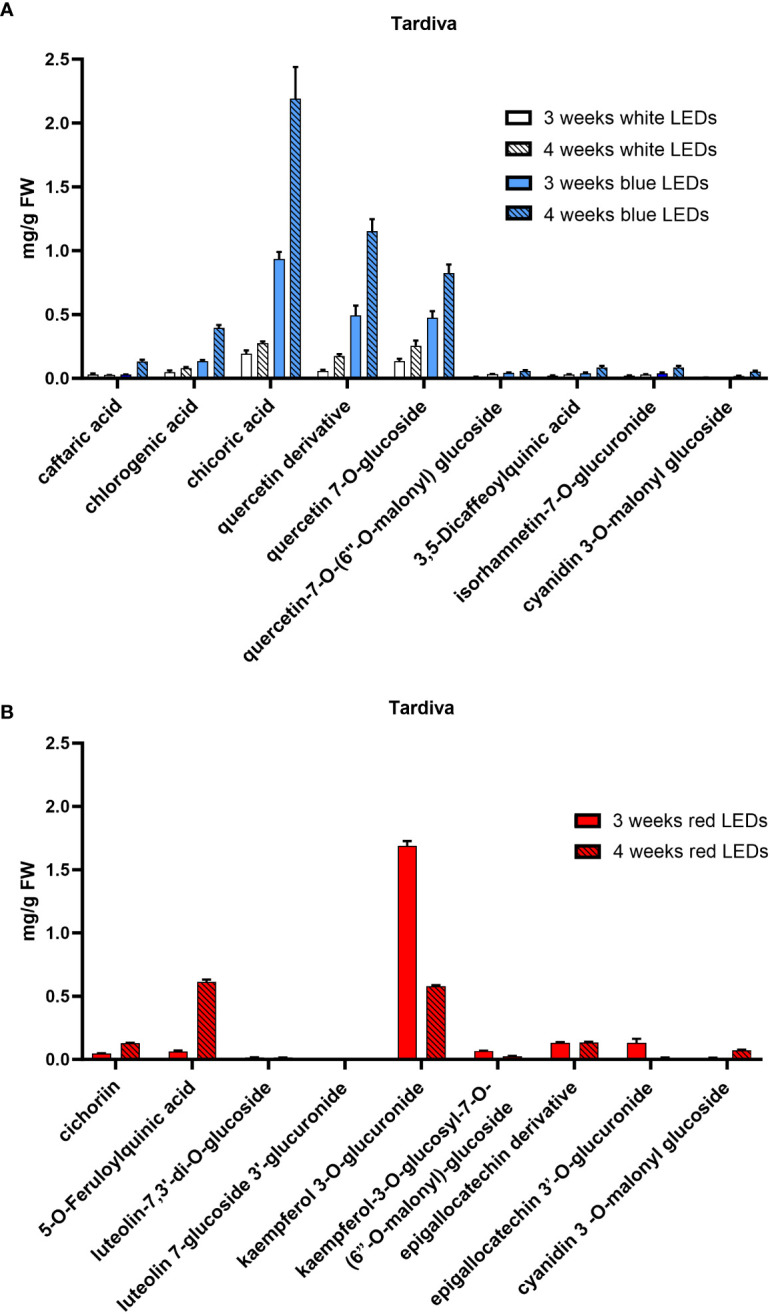
Polyphenolic composition of *C*. *intybus* Tardiva cultivar after three and four weeks of treatment with white, blue **(A)** and red **(B)** LEDs.

**Figure 6:**
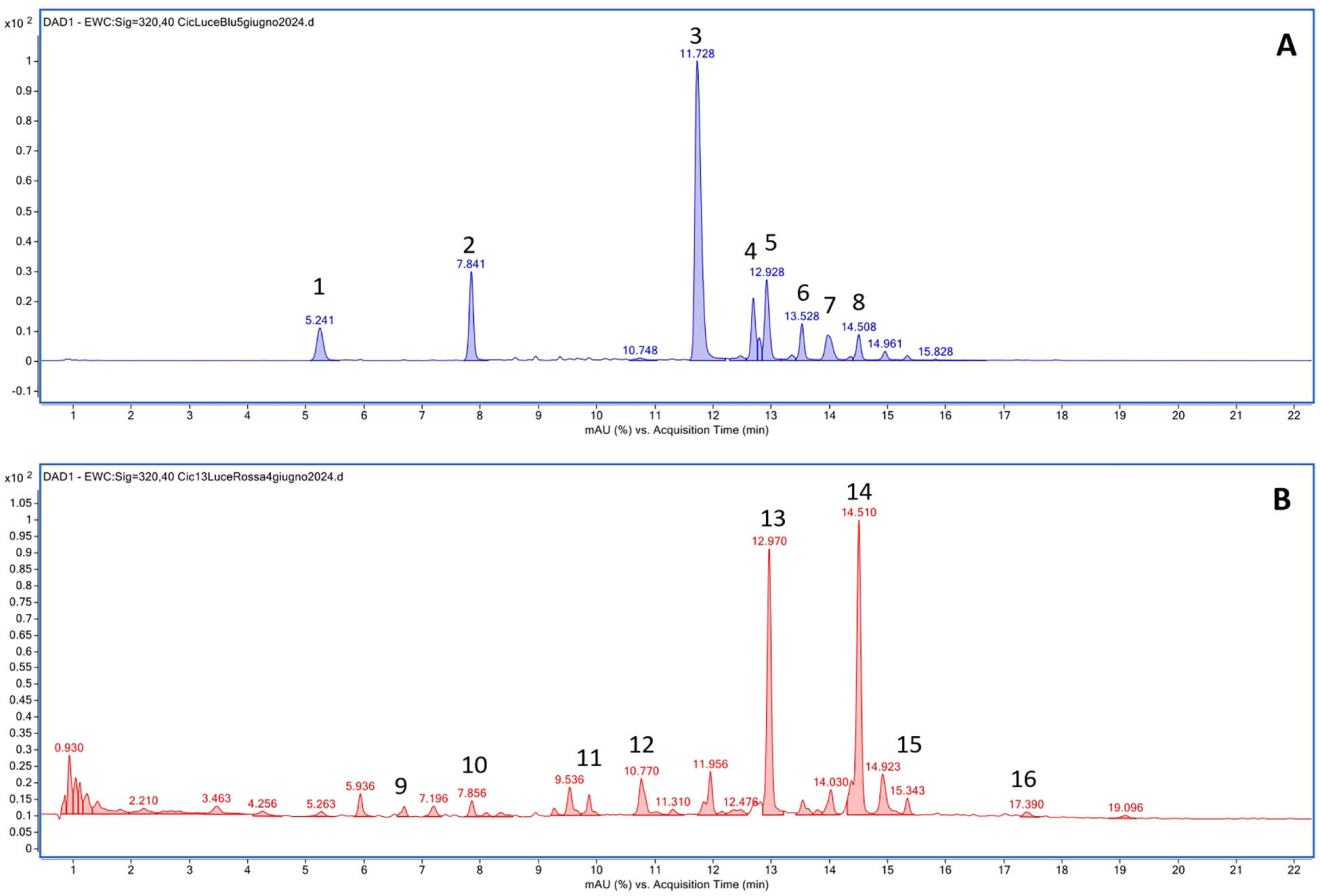
Representative chromatograms obtained by HPLC-DAD-MS analysis for *C*. *intybus* Tardiva cultivar after the treatment with blue **(A)** and red **(B)** LEDs. 1: Caftaric acid; 2: Chlorogenic acid; 3: Chicoric acid; 4: Quercetin derivative; 5: Quercetin-7-O-glucoside; 6: Quercetin-7-O-(6’’-O-malonyl) glucoside; 7: 3,5-Di-caffeoylquinic acid; 8: Isorhamnetin-7-O-glucuronide; 9: Cichoriin; 10: 5-O-Feruloylquinic acid; 11: Luteolin-7,3’-di-O-glucoside; 12: Luteolin 7-glucoside 3’-glucuronide; 13: Kaempferol 3-O-glucuronide; 14: Kaempferol-3-O-glucosyl-7-O-(6”-O-malonyl)-glucoside; 15: Epigallocatechin derivative; 16: Epigallocatechin 3’-O-glucuronide.

The HPLC-DAD-MS analysis allowed the identification of the polyphenols in the two cultivars (**Table 1**) and revealed differences in the composition depending on the light treatment supplied and, to a lesser extent, on the considered cultivar. In both cultivars, a similar composition was observed in plants grown under white and blue LEDs, and a time-dependent accumulation was observed for all compounds. Under white and blue LEDs, 12 polyphenolic compounds were detected in the Precoce (**Supplementary Table S1**, **Figure 4A**). The same composition was observed in Tardiva, excepting for the absence of kuromanin and three cyanidin derivatives, and the presence of isorhamnetin-7-O-glucuronide (**Supplementary Table S2**, **Figures 5A**, **6A**).

**Table 1:** UV-vis, MS and MS^2^ data of the polyphenolic compounds detected in *C. intybus* Precoce and Tardiva cultivars in response to white, blue and red LEDs-treatments.

			ESI(-)-QToF/MS					Assignment	
Compound no	LC Rt (min)	DAD max abs (nm)	Precursor ion (m/z)	Adducts and fragment Ions	Exp. Acc. Mass [M - H]-	Detected mass	Diff (ppm)	Tentative Identification	Molecular formula
1	5.39	330, 300 sh	311.04	135.04	311.0416	311.0416	1.08	Caftaric acid	C13H12O9
2	7.94	325, 295 sh	353.08	191.05	353.0878	353.0909	7.82	Chlorogenic acid	C16H18O9
3	11.81	325	473.07	179.03; 149.00	473.0725	473.0703	-5.46	Chicoric acid	C22H18O12
4	12.78	355, 300sh	477.05	301; 151	477.0675	477.0677	-1.23	Quercetin derivative	C21H18O13
5	12.95	350	463.08	300.02	463.0882	463.0890	1.38	Quercetin-7-O-glucoside	C21H20O12
6	13.86	355, 300sh	549.08	300.03	549.0886	549.0893	1.39	Quercetin-7-O-(6’’-O-malonyl) glucoside	C24H22O15
7	14.09	330, 295 sh	515.11	173.04;191.05	515.1195	515.1150	-6.33	3,5-Di caffeoylquinic acid	C25H24O12
8	14.61	340	491.08	300.02; 315.04;271.02	491.0831	491.0792	-8.45	Isorhamnetin-7-O-glucuronide	C22H20O13
9	6.78	335, 290	339.07	177.01	339.0722	339.0724	0.86	Cichoriin	C15H16O9
10	7.97	330,290	367.10	235.11; 367.16	367.1035	–	-	5-O-Feruloylquinic acid	C17H20O9
11	9.99	345, 270	609.14	447.09;285.03	609.1461	609.1417	2.39	Luteolin-7,3’-di-O-glucoside	C27H30O16
12	10.9	338, 270	623.12	285.04; 461.07	623.1254	623.1275	3.35	Luteolin 7-glucoside 3’-glucuronide	C27H28O17
13	13.08	350, 270 sh 255 sh	461.03	285.02	461.0725	461.0733	1.46	Kaempferol 3-O-Glucuronide	C21H18O12
14	14.45	350,285	695.28	303.14;161.04;101.02	695.2768	695.2808	5.08	Kaempferol-3-O-glucosyl-7-O-(6”-O-malonyl)-glucoside	C30H48O18
17	16.97	275	489.10	445.2	489.0886	489.0863	-4.59	Kaempferol-7-O-(6’’-O-acetyl)-glucoside	C19H22O15
15	15.44	355, 270 sh	433.20	–	433.2079	433.2090	2.51	Epigallochatechin derivative	C20H34O10
16	17.56	265	481.11	213.09;257.08	481.0988	481.1163	–	Epigallocatechin 3’-O-glucuronide	C21H22O13

The red LEDs induced an accumulation of nine compounds in both cultivars (**Supplementary Tables S1**, **S2**). The Precoce also showed the presence of two additional compounds, *i.e.*, kuromanin and cyanin 3,5 diglucoside (**Figure 4B**).

Chicoric acid, followed by quercetin derivative and quercetin 7-O-glucoside, were the main polyphenolic compounds detected in both cultivars exposed to white and blue LEDs. Kaempferol 3-O-glucuronide was the most represented compound found in plants exposed to red LEDs, and in the case of Tardiva cultivar, also high levels of 5-O-feruloylquinic acid were observed after four weeks of treatment (**Figures 5B**, **6B**).

## 4 Discussion

In greenhouse horticulture, LEDs enable precise manipulation of light spectral composition, which makes them effective tools for optimizing both crop production and quality (Paradiso and Proietti, 2022). Light treatments using precisely tuned red and blue wavelengths are known to enhance the secondary metabolite content in plants, including polyphenols (Taulavuori et al., 2018; Alrifai et al., 2019; Loi et al., 2020). However, since specific responses may vary among species and genotypes, determining the optimal combination of cultivar and light treatment is crucial for achieving the best composition and highest accumulation of useful bioactive compounds (Gómez and Izzo, 2018; Taulavuori et al., 2018). This study elucidated the impact of different LEDs on the phenolic composition of two *C. intybus* cultivars, thus allowing the selection of the most promising light treatment to improve the content of antioxidant polyphenols.

### 4.1 Effect of the light treatments on the PS II efficiency, the content of chlorophylls and the content of total polyphenols

PS II efficiency was negatively affected by the red LEDs treatment in both cultivars, whereas the performances of the PS II did not vary in response to white and blue LEDs exposure. It is worth noting that longer wavelengths, such as those in the red and far-red region of the light spectrum, preferentially excite PS I and when a PS is overexcited the efficiency of the other one is limited (Zhen et al., 2019). An overexcitation of the PS I caused by the red treatment could explain the lower efficiency values observed in both *C. intybus* cultivars with respect to the other treatments. Nevertheless, it has been observed in other species, such as potato (Chen et al., 2021) and *Artemisia argyi* (Su et al., 2024) that plants grown under monochromatic red light develop a reduced potential photosynthetic capability in PS II and an increased light energy dissipation *via* non-photochemical quenching, compared to plants grown under white or blue light.

Concerning the chlorophyll content, the highest accumulation was induced by blue LEDs in both cultivars. Particularly, in the Precoce, chlorophyll accumulation was directly proportional to the time, also highlighting a clear distinction between the effect of blue LEDs and that of the other two irradiance systems at a more mature stage of development of the leaf (*i.e.*, 4 weeks).

The blue LEDs also determined the greatest accumulation of polyphenols in both cultivars, at the early and mature state of leaf development. Similar increases in total chlorophyll content and polyphenols levels induced by blue LEDs light were previously observed in other herbaceous species (Johkan et al., 2010; Manivannan et al., 2015; Lobiuc et al., 2017; Park et al., 2019; Azad et al., 2020). Particularly, under blue LEDs, the polyphenols content in the Tardiva was more than doubled at four weeks, pointing out the correlation between the accumulation of these metabolites and leaf development. This is in agreement with previous studies demonstrating that blue LEDs-irradiation constitutes a powerful tool to produce high-quality red leaf vegetables, leading to an improved content of bioprotective pigments (Son and Oh, 2013; Carvalho and Folta, 2014).

Both blue and red lights are efficiently absorbed by plant photosynthetic pigments, but the blue wavelength contains more energy (Ouzounis et al., 2015; Gómez and Izzo, 2018; Sytar et al., 2019). Among all monochromatic radiations, the red one is the most efficiently converted into chemical energy, hence resulting in very photosynthetically active radiation (Gómez and Izzo, 2018). Tardiva in fact positively reacted also to the treatment with red LED, exhibiting a significantly higher polyphenols content than plants exposed to white LEDs. This increase was stable over time, although considerably lower compared to that observed for blue light. Research on LEDs manipulation already showed that the responses to light quality are species-specific, requiring unique protocols to obtain high-productive and high-nutritive vegetables, since a great variability of responses can be observed depending on the lighting conditions (*i.e.*, light wavelength, intensity, photoperiod) (Mitchell and Stutte, 2015; Gómez and Izzo, 2018; Appolloni et al., 2022; Orlando et al., 2022; da Cristina Bungala et al., 2024). In some cases, responses can be different in diverse cultivars within the same species (Gómez and Izzo, 2018). In the case of chicory, our results suggested that the main differences seemed not to be correlated to the cultivars, but rather to the type of light provided during plants growth. Overall, the best performances in terms of both chlorophyll and polyphenols accumulation were obtained under blue LEDs. In terms of quantities, the Precoce showed the greatest content of polyphenolic compounds, regardless of the type of LEDs used for its cultivation. Most of the studies on microgreens, including those conducted on chicory (Pennisi et al., 2020), explore the effect of blue and red LEDs combined in different ratios, but very different results have been reported, especially regarding metabolite accumulation (Appolloni et al., 2022). In some cases, the best performances were obtained with monochromatic light only (Zhang et al., 2019), suggesting that the lighting system must be carefully tuned according to the species responses, in order to optimize the production.

### 4.2 Effect of blue, red and white LEDs on the polyphenolic composition

The polyphenolic composition of the two *C. intybus* cultivars was strongly influenced by the type of light used for their cultivation. For plants grown under white and blue LEDs, only quantitative differences were found in the leaf polyphenols, whereas a completely different composition characterized the leaf extracts of those plants grown under red LEDs. This pattern was consistent across both cultivars (**Supplementary Figure S4**). When exposed to white/blue LEDs, the leaves accumulated polyphenolic compounds with high antioxidant potential, such as quercetin derivatives and chicoric acid (Lesjak et al., 2018). This aligns with the increased efficiency exhibited by the PS II in response to white/blue LEDs and the higher content of chlorophyll as well, suggesting an intense photosynthetic activity. In such conditions, the disposal of strong antioxidant compounds ensures the prevention of ROS-related damages, being ROS massively produced during photosynthesis (Foyer, 2018; Singh et al., 2021).

The biosynthesis of quercetin is catalyzed by flavonol synthase (FLS), an enzyme that is affected by light spectral composition (Singh et al., 2021). For example, it was observed that the expression of *LsFTS* gene coding for the FLS in lettuce, was considerably enhanced by the combined irradiation with UV-B plus blue light compared to UV-B radiation only. This corresponded to a significant increase in the leaf quercetin (Ebisawa et al., 2008). Flavonol accumulation induced by blue LEDs has also been reported in other species (Li et al., 2018; Wang et al., 2022). A similar effect could be hypothesized for the two *C. intybus* cultivars considered in this study, as the metabolic pathway for the biosynthesis of flavonols is very ancient and highly conserved (Pollastri and Tattini, 2011). Particularly, in our study, it is likely that the enhancement of both FLS and F3’H (Flavonoid 3’-hydroxylase) activity induced by blue LEDs led to the accumulation of quercetin derivatives rather than kaempferol derivatives (Shi et al., 2014). In particular, blue LEDs enhanced the accumulation of three different quercetin derivatives (quercetin derivative, quercetin 7-O-glucoside, quercetin-7-O-(6’’-O-malonyl) glucoside), all present in the control (white LEDs), but in lower amounts. By enhancing the activity of several enzymes involved in the polyphenols biosynthesis, and in particular of the above-mentioned FLS and F3’H, blue light induces the accumulation of functional compounds with nutraceutical properties in many horticultural crops (Heo et al., 2012; Alrifai et al., 2019; Orlando et al., 2022).

In contrast, red LEDs drove the flavonoid biosynthetic pathway towards the accumulation of kaempferols, with kaempferol 3-O-glucuronide being predominant in both cultivars (**Supplementary Figure S4**). Due to their structure (i.e. lower number of hydroxyl groups and lack of catechol in the molecule), these compounds are less antioxidant than quercetins (Rice-Evans et al., 1996; Agati and Tattini, 2010; Dueñas et al., 2011). This structural difference supports the hypothesis that red LEDs irradiance may induce lower stress levels in plants, as also confirmed by a reduced amount of caffeic acid derivatives in favor of ferulic acid derivatives which have a more structural property for cell walls rather than antioxidant activity (Harris and Trethewey, 2010). Accordingly, only few anthocyanins, which are also known to play a protective role as antioxidants and ROS scavengers (Quina et al., 2009; Tena et al., 2020; Agati et al., 2021), were found in Precoce seedlings treated with red LEDs compared to those exposed to blue LEDs. This result is in accordance with the presence of epicatechin derivatives detected only under red light, suggesting an induction of ANR (anthocyanidin reductase) by red light (Zhang et al., 2018). Monochromatic red light was found to prevent the synthesis of anthocyanins in red curly lettuce (Heo et al., 2012), whereas there is good evidence that blue LEDs typically induces an accumulation of anthocyanins in many horticultural plants, even at the postharvest phase (Johkan et al., 2010; Xu et al., 2014; Sytar et al., 2019). For instance, Stutte et al., 2009 evaluated the effect of different LEDs on red leaf lettuce, finding that after exposure to blue LEDs, the content of bioprotective anthocyanins was more than doubled compared to what observed under red LEDs.

## 5 Conclusions

Blue LEDs irradiance induced a time-dependent accumulation of leaf polyphenols and chlorophylls in seedlings of *C. intybus* belonging to Precoce and Tardiva cultivars, favoring the biosynthesis of highly antioxidant compounds (*e.g.*, quercetin derivatives and chicoric acid) compared to red LEDs. The Precoce cultivar exhibited the highest content of polyphenols, regardless of the type of LEDs used for cultivation. These findings suggest that blue LEDs could be effectively used as a tool for improving the nutraceutical content of *C. intybus*, especially for the Precoce cultivar.

## Data Availability

The original contributions presented in the study are included in the article/**Supplementary Material**. Further inquiries can be directed to the corresponding authors.

## References

[B1] Agati G. Guidi L. Landi M. Tattini M. (2021). Anthocyanins in photoprotection: knowing the actors in play to solve this complex ecophysiological issue. New Phytol. 232, 2228–2235. doi: 10.1111/nph.17648 34449083 PMC9291080

[B2] Agati G. Tattini M. (2010). Multiple functional roles of flavonoids in photoprotection. New Phytol. 186, 786–793. doi: 10.1111/j.1469-8137.2010.03269.x 20569414

[B3] Aldahak L. Salem K. F. Al-Salim S. H. Al-Khayri J. M. (2021). Advances in chicory (*Cichorium intybus* l.) breeding strategies. Adv. Plant Breed. strategies: vegetable crops: volume 10: leaves flowerheads Green pods mushrooms truffles, 3–57. doi: 10.1007/978-3-030-66969-0_1

[B4] Alrifai O. Hao X. Marcone M. F. Tsao R. (2019). Current review of the modulatory effects of LED lights on photosynthesis of secondary metabolites and future perspectives of microgreen vegetables. J. Agric. Food Chem. 67, 6075–6090. doi: 10.1021/acs.jafc.9b00819 31021630

[B5] Appolloni E. Pennisi G. Zauli I. Carotti L. Paucek I. Quaini S.. (2022). Beyond vegetables: effects of indoor LED light on specialized metabolite biosynthesis in medicinal and aromatic plants, edible flowers, and microgreens. J. Sci. Food Agric. 102, 472–487. doi: 10.1002/jsfa.v102.2 34462916 PMC9292972

[B6] Azad M. O. K. Kjaer K. H. Adnan M. Naznin M. T. Lim J. D. Sung I. J.. (2020). The evaluation of growth performance, photosynthetic capacity, and primary and secondary metabolite content of leaf lettuce grown under limited irradiation of blue and red LED light in an urban plant factory. Agriculture 10, 28. doi: 10.3390/agriculture10020028

[B7] Carazzone C. Mascherpa D. Gazzani G. Papetti A. (2013). Identification of phenolic constituents in red chicory salads (*Cichorium intybus*) by high-performance liquid chromatography with diode array detection and electrospray ionisation tandem mass spectrometry. Food Chem. 138, 1062–1071. doi: 10.1016/j.foodchem.2012.11.060 23411215

[B8] Carvalho S. D. Folta K. M. (2014). Sequential light programs shape kale (Brassica napus) sprout appearance and alter metabolic and nutrient content. Horticulture Res. 1, 2014, 8. doi: 10.1038/hortres.2014.8 PMC4591675 26504531

[B9] Chen L. L. Wang H. Y. Gong X. C. Zeng Z. H. Xue X. Z. Hu Y. G. (2021). Transcriptome analysis reveals effects of red and blue light-emitting diodes (LEDs) on the growth, chlorophyll fluorescence and endogenous plant hormones of potato (Solanum tuberosum L.) plantlets cultured in *vitro* . J. Integr. Agric. 20, 2914–2931. doi: 10.1016/S2095-3119(20)63393-7

[B10] D’evoli L. Morroni F. Lombardi-Boccia G. Lucarini M. Hrelia P. Cantelli-Forti G.. (2013). Red chicory (*Cichorium intybus* L. cultivar) as a potential source of antioxidant anthocyanins for intestinal health. Oxid. Med. Cell. Longevity 2013, 704310. doi: 10.1155/2013/704310 PMC3771420 24069504

[B11] da Cristina Bungala L. T. Park S. U. Van Nguyen B. Lim J. Kim K. Kim J. K.. (2024). Effect of LED lights on secondary metabolites and antioxidant activities in red pakchoi baby leaves. ACS omega 9, 23420. doi: 10.1021/acsomega.3c10261 38854528 PMC11154946

[B12] Dueñas M. Surco-Laos F. González-Manzano S. González-Paramás A. M. Santos-Buelga C. (2011). Antioxidant properties of major metabolites of quercetin. Eur. Food Res. Technol. 232, 103–111. doi: 10.1007/s00217-010-1363-y

[B13] Ebisawa M. Shoji K. Kato M. Shimomura K. Goto F. Yoshihara T. (2008). Supplementary ultraviolet radiation B together with blue light at night increased quercetin content and flavonol synthase gene expression in leaf lettuce (Lactuca sativa L.). Environ. Control Biol. 46, 1–11. doi: 10.2525/ecb.46.1

[B14] Foyer C. H. (2018). Reactive oxygen species, oxidative signaling and the regulation of photosynthesis. Environ. Exp. Bot. 154, 134–142. doi: 10.1016/j.envexpbot.2018.05.003 30283160 PMC6105748

[B15] Gómez C. Izzo L. G. (2018). Increasing efficiency of crop production with LEDs. AIMS Agric. Food 3, 135–153. doi: 10.3934/agrfood.2018.2.135

[B16] Harris P. J. Trethewey J. A. (2010). The distribution of ester-linked ferulic acid in the cell walls of angiosperms. Phytochem. Rev. 9, 19–33. doi: 10.1007/s11101-009-9146-4

[B17] Heo J. W. Kang D. H. Bang H. S. Hong S. G. Chun C. Kang K. K. (2012). Early growth, pigmentation, protein content, and phenylalanine ammonia-lyase activity of red curled lettuces grown under different lighting conditions. Kor. J. Hortic. Sci. Technol. 30, 6–12. doi: 10.7235/hort.2012.11118

[B18] Iqbal Y. Ponnampalam E. N. Suleria H. A. Cottrell J. J. Dunshea F. R. (2021). LC-ESI/QTOF-MS profiling of chicory and lucerne polyphenols and their antioxidant activities. Antioxidants 10, 932. doi: 10.3390/antiox10060932 34201340 PMC8226608

[B19] Islam M. A. Kuwar G. Clarke J. L. Blystad D. R. Gislerød H. R. Olsen J. E.. (2012). Artificial light from light emitting diodes (LEDs) with a high portion of blue light results in shorter poinsettias compared to high pressure sodium (HPS) lamps. Scientia Hortic. 147, 136–143. doi: 10.1016/j.scienta.2012.08.034

[B20] Janda K. Gutowska I. Geszke-Moritz M. Jakubczyk K. (2021). The common cichory (*Cichorium intybus* L.) as a source of extracts with health-promoting properties—a review. Molecules 26, 1814. doi: 10.3390/molecules26061814 33807029 PMC8005178

[B21] Johkan M. Shoji K. Goto F. Hashida S. N. Yoshihara T. (2010). Blue light-emitting diode light irradiation of seedlings improves seedling quality and growth after transplanting in red leaf lettuce. HortScience 45, 1809–1814. doi: 10.21273/HORTSCI.45.12.1809

[B22] Kalantari F. Tahir O. M. Joni R. A. Fatemi E. (2018). Opportunities and challenges in sustainability of vertical farming: A review. J. Landscape Ecol. 11, 35–60. doi: 10.1515/jlecol-2017-0016

[B23] Kiani H. S. Ahmad W. Nawaz S. Farah M. A. Ali A. (2023). Optimized extraction of polyphenols from unconventional edible plants: LC-MS/MS profiling of polyphenols, biological functions, molecular docking, and pharmacokinetics study. Molecules 28, 6703. doi: 10.3390/molecules28186703 37764478 PMC10534510

[B24] Kim D. H. Jeong D. K. Kim H. Chon J. W. Lim H. W. Chang H. S.. (2017). Manufacture of functional koumiss supplemented with *Cichorium intybus* L.(chicory) extract-preliminary study. J. Dairy Sci. Biotechnol. 35, 1–7. doi: 10.22424/jmsb.2017.35.1.001

[B25] Lesjak M. Beara I. Simin N. Pintać D. Majkić T. Bekvalac K.. (2018). Antioxidant and anti-inflammatory activities of quercetin and its derivatives. J. Funct. Foods 40, 68–75. doi: 10.1016/j.jff.2017.10.047

[B26] Li H. Lin Y. Chen X. Bai Y. Wang C. Xu X.. (2018). Effects of blue light on flavonoid accumulation linked to the expression of miR393, miR394 and miR395 in longan embryogenic calli. PloS One 13, e0191444. doi: 10.1371/journal.pone.0191444 29381727 PMC5790225

[B27] Lobiuc A. Vasilache V. Pintilie O. Stoleru T. Burducea M. Oroian M.. (2017). Blue and red LED illumination improves growth and bioactive compounds contents in acyanic and cyanic Ocimum basilicum L. microgreens. Molecules 22, 2111. doi: 10.3390/molecules22122111 29189746 PMC6150032

[B28] Loi M. Villani A. Paciolla F. Mulè G. Paciolla C. (2020). Challenges and opportunities of light-emitting diode (LED) as key to modulate antioxidant compounds in plants. A review. Antioxidants 10, 42. doi: 10.3390/antiox10010042 33396461 PMC7824119

[B29] Manivannan A. Soundararajan P. Halimah N. Ko C. H. Jeong B. R. (2015). Blue LED light enhances growth, phytochemical contents, and antioxidant enzyme activities of Rehmannia glutinosa cultured in *vitro* . Horticulture Environment Biotechnol. 56, 105–113. doi: 10.1007/s13580-015-0114-1

[B30] Martínez-Ispizua E. Calatayud Á. Marsal J. I. Cannata C. Basile F. Abdelkhalik A.. (2022). The nutritional quality potential of microgreens, baby leaves, and adult lettuce: an underexploited nutraceutical source. Foods 11, 423. doi: 10.3390/foods11030423 35159573 PMC8834567

[B31] Mir M. S. Naikoo N. B. Kanth R. H. Bahar F. A. Bhat M. A. Nazir A.. (2022). Vertical farming: The future of agriculture: A review. Pharma Innovation J. 11, 1175–1195.

[B32] Mitchell C. Stutte G. (2015). “Sole-source lighting for controlled-environment agriculture,” in Light Management in Controlled Environments. Eds. Lopez R. Runkle E. S.. (Lopez R. Runkle E., Meister Media Worldwide Acquisition Source: Kennedy Space Center), 119–134.

[B33] Orlando M. Trivellini A. Incrocci L. Ferrante A. Mensuali A. (2022). The inclusion of green light in a red and blue light background impact the growth and functional quality of vegetable and flower microgreen species. Horticulturae 8, 217. doi: 10.3390/horticulturae8030217

[B34] Ouzounis T. Rosenqvist E. Ottosen C. O. (2015). Spectral effects of artificial light on plant physiology and secondary metabolism: A review. HortScience 50, 1128–1135. doi: 10.21273/HORTSCI.50.8.1128

[B35] Papetti A. Maietta M. Corana F. Marrubini G. Gazzani G. (2017). Polyphenolic profile of green/red spotted Italian *Cichorium intybus* salads by RP-HPLC-PDA-ESI-MSn. J. Food Composition Anal. 63, 189–197. doi: 10.1016/j.jfca.2017.08.010

[B36] Paradiso R. Proietti S. (2022). Light-quality manipulation to control plant growth and photomorphogenesis in greenhouse horticulture: the state of the art and the opportunities of modern LED systems. J. Plant Growth Regul. 41, 742–780. doi: 10.1007/s00344-021-10337-y

[B37] Park C. H. Kim N. S. Park J. S. Lee S. Y. Lee J. W. Park S. U. (2019). Effects of light-emitting diodes on the accumulation of glucosinolates and phenolic compounds in sprouting canola (Brassica napus L.). Foods 8, 76. doi: 10.3390/foods8020076 30791403 PMC6406741

[B38] Pennisi G. Orsini F. Landolfo M. Pistillo A. Crepaldi A. Nicola S.. (2020). Optimal photoperiod for indoor cultivation of leafy vegetables and herbs. Eur. J. Hortic. Sci. 85, 329–338. doi: 10.17660/eJHS.2020/85.5.4

[B39] Pennisi G. Sanyé-Mengual E. Orsini F. Crepaldi A. Nicola S. Ochoa J.. (2019). Modelling environmental burdens of indoor-grown vegetables and herbs as affected by red and blue LED lighting. Sustainability 11, 4063. doi: 10.3390/su11154063

[B40] Pollastri S. Tattini M. (2011). Flavonols: old compounds for old roles. Ann. Bot. 108, 1225–1233. doi: 10.1093/aob/mcr234 21880658 PMC3197460

[B41] Quina F. H. Moreira P. F. Jr. Vautier-Giongo C. Rettori D. Rodrigues R. F. Freitas A. A.. (2009). Photochemistry of anthocyanins and their biological role in plant tissues. Pure Appl. Chem. 81, 1687–1694. doi: 10.1351/PAC-CON-08-09-28

[B42] Rice-Evans C. A. Miller N. J. Paganga G. (1996). Structure-antioxidant activity relationships of flavonoids and phenolic acids. Free Radical Biol. Med. 20, 933–956. doi: 10.1016/0891-5849(95)02227-9 8743980

[B43] Shi L. Cao S. Chen W. Yang Z. (2014). Blue light induced anthocyanin accumulation and expression of associated genes in Chinese bayberry fruit. Scientia Hortic. 179, 98–102. doi: 10.1016/j.scienta.2014.09.022

[B44] Singh P. Arif Y. Bajguz A. Hayat S. (2021). The role of quercetin in plants. Plant Physiol. Biochem. 166, 10–19. doi: 10.1016/j.plaphy.2021.05.023 34087741

[B45] Son K. H. Oh M. M. (2013). Leaf shape, growth, and antioxidant phenolic compounds of two lettuce cultivars grown under various combinations of blue and red light-emitting diodes. HortScience 48, 988–995. doi: 10.21273/HORTSCI.48.8.988

[B46] Stutte G. W. Edney S. Skerritt T. (2009). Photoregulation of bioprotectant content of red leaf lettuce with light-emitting diodes. HortScience 44, 79–82. doi: 10.21273/HORTSCI.44.1.79

[B47] Su P. Ding S. Wang D. Kan W. Yuan M. Chen X.. (2024). Plant morphology, secondary metabolites and chlorophyll fluorescence of Artemisia argyi under different LED environments. Photosynthesis Res. 159, 153–164. doi: 10.1007/s11120-023-01026-w PMC10197053 37204684

[B48] Sytar O. Zivcak M. Neugart S. Toutounchi P. M. Brestic M. (2019). Precultivation of young seedlings under different color shades modifies the accumulation of phenolic compounds in Cichorium leaves in later growth phases. Environ. Exp. Bot. 165, 30–38. doi: 10.1016/j.envexpbot.2019.05.018

[B49] Taulavuori K. Pyysalo A. Taulavuori E. Julkunen-Tiitto R. (2018). Responses of phenolic acid and flavonoid synthesis to blue and blue-violet light depends on plant species. Environ. Exp. Bot. 150, 183–187. doi: 10.1016/j.envexpbot.2018.03.016

[B50] Tena N. Martín J. Asuero A. G. (2020). State of the art of anthocyanins: Antioxidant activity, sources, bioavailability, and therapeutic effect in human health. Antioxidants 9, 451. doi: 10.3390/antiox9050451 32456252 PMC7278599

[B51] Tsuda T. (2012). Dietary anthocyanin-rich plants: biochemical basis and recent progress in health benefits studies. Mol. Nutr. Food Res. 56, 159–170. doi: 10.1002/mnfr.201100526 22102523

[B52] Wallace T. C. (2011). Anthocyanins in cardiovascular disease. Adv. Nutr. 2, 1–7. doi: 10.3945/an.110.000042 22211184 PMC3042791

[B53] Wang G. Zhang L. Wang G. Cao F. (2022). Growth and flavonol accumulation of Ginkgo biloba leaves affected by red and blue light. Ind. Crops Products 187, 115488. doi: 10.1016/j.indcrop.2022.115488

[B54] Xu F. Cao S. Shi L. Chen W. Su X. Yang Z. (2014). Blue light irradiation affects anthocyanin content and enzyme activities involved in postharvest strawberry fruit. J. Agric. Food Chem. 62, 4778–4783. doi: 10.1021/jf501120u 24783962

[B55] Zhang X. Bian Z. Li S. Chen X. Lu C. (2019). Comparative analysis of phenolic compound profiles, antioxidant capacities, and expressions of phenolic biosynthesis-related genes in soybean microgreens grown under different light spectra. J. Agric. Food Chem. 67, 13577–13588. doi: 10.1021/acs.jafc.9b05594 31730344

[B56] Zhang Y. Jiang L. Li Y. Chen Q. Ye Y. Zhang Y.. (2018). Effect of red and blue light on anthocyanin accumulation and differential gene expression in strawberry (Fragaria× ananassa). Molecules 23, 820. doi: 10.3390/molecules23040820 29614032 PMC6017741

[B57] Zhen S. Haidekker M. van Iersel M. W. (2019). Far-red light enhances photochemical efficiency in a wavelength-dependent manner. Physiologia plantarum 167, 21–33. doi: 10.1111/ppl.v167.1 30203475

