# Supplementary figures and images for: Blue and red LEDs modulate polyphenol production in Precoce and Tardiva cultivars of Cichorium intybus L
Source: Front Plant Sci. 2025 Feb 21;16:1529804. doi: 10.3389/fpls.2025.1529804 (PMC11885293; doi:10.3389/fpls.2025.1529804)

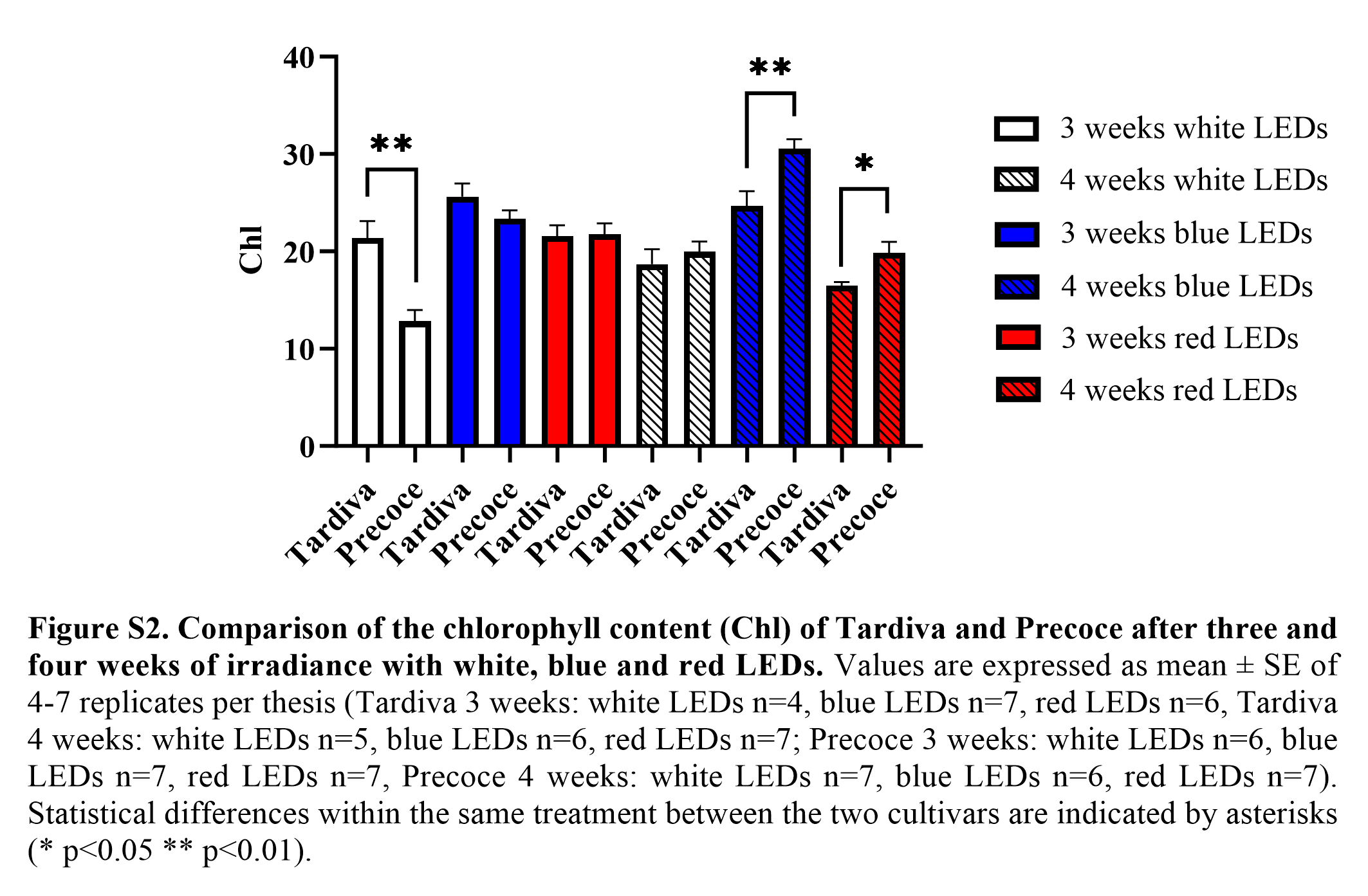

Supplement: Supplementary file 1 [file Image1.tif]

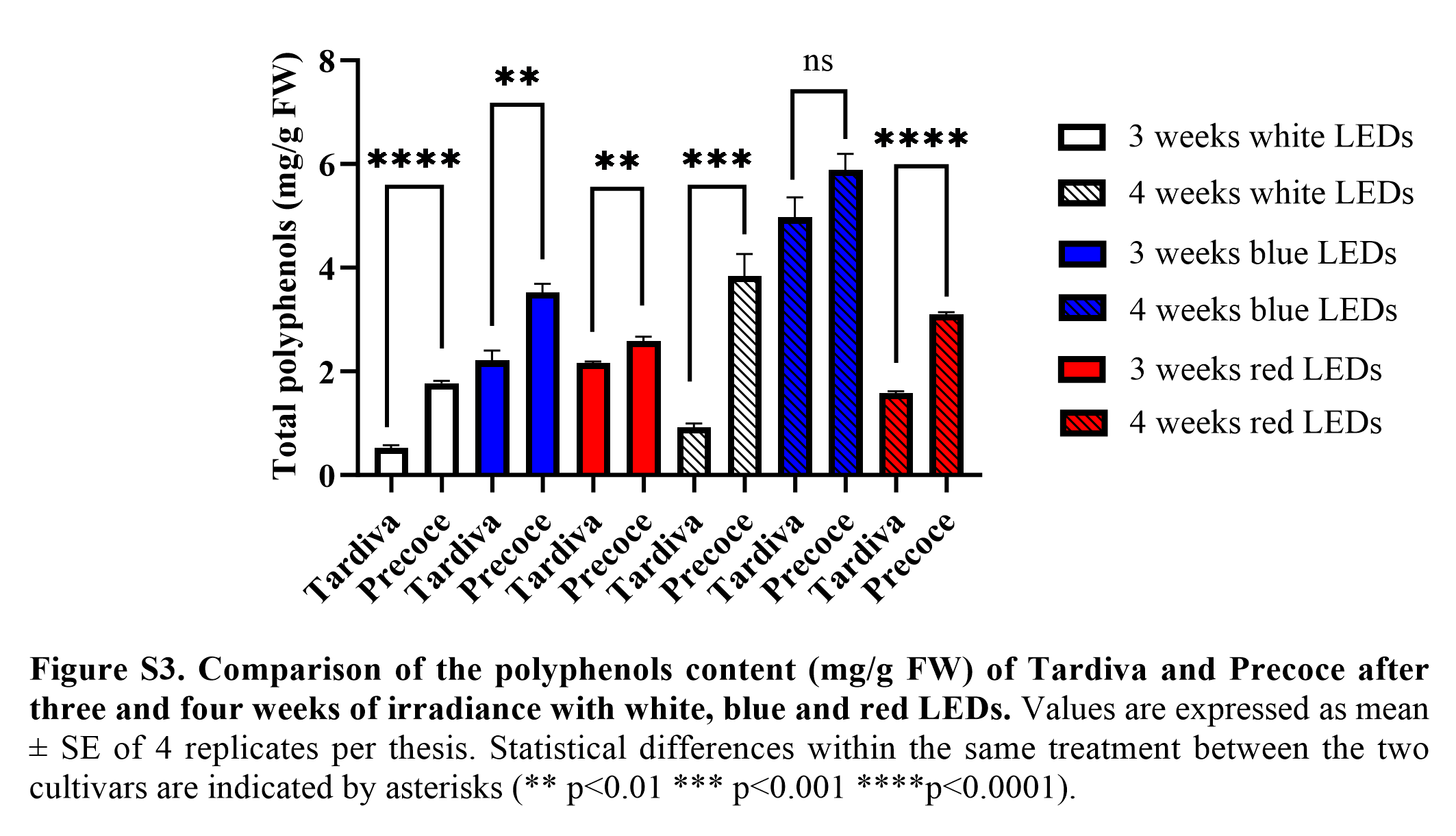

Supplement: Supplementary file 2 [file Image2.tif]

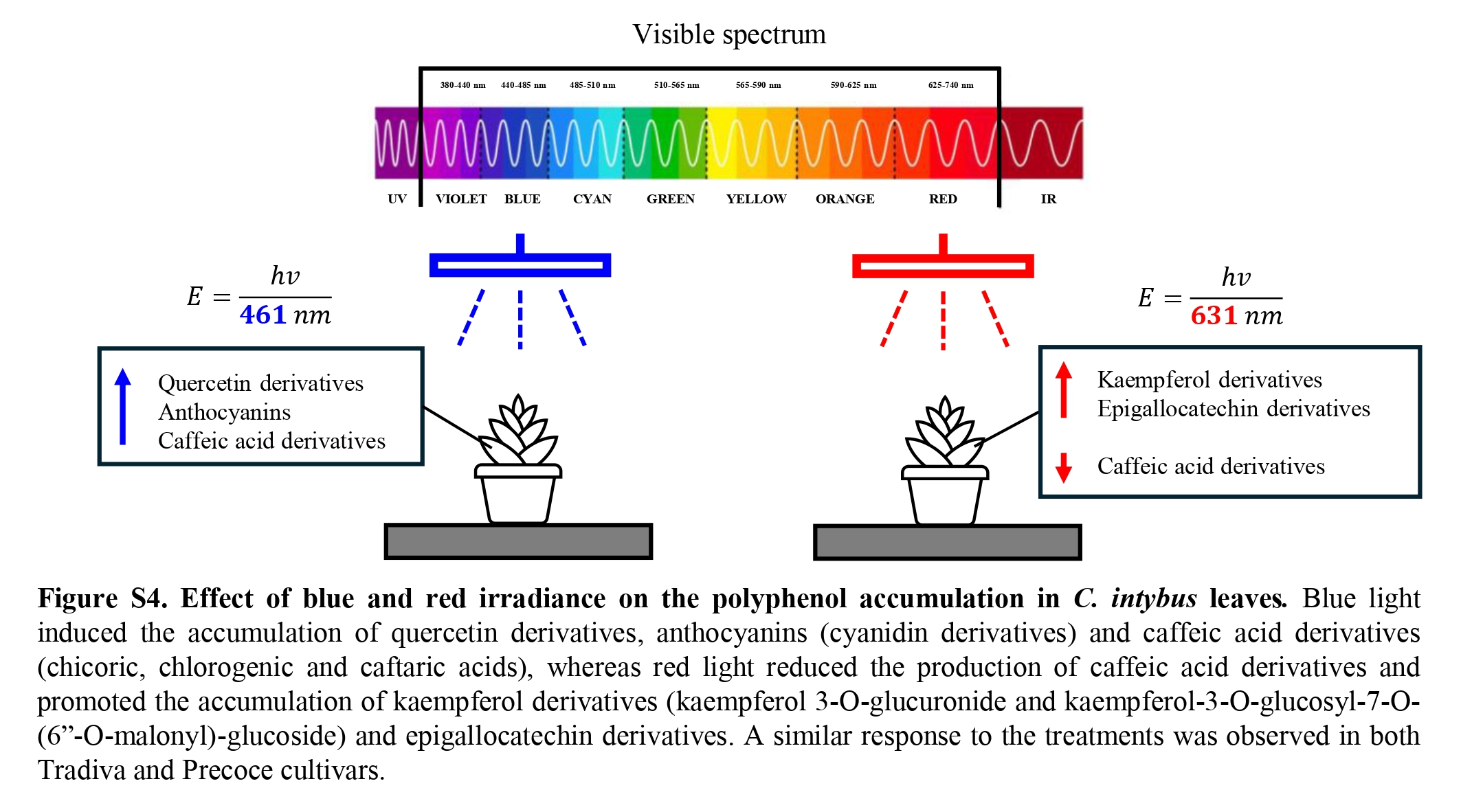

Supplement: Supplementary file 3 [file Image3.jpeg]
