# Supplementary material for: Blue and red LEDs modulate polyphenol production in Precoce and Tardiva cultivars of Cichorium intybus L
Source: Front Plant Sci. 2025 Feb 21;16:1529804. doi: 10.3389/fpls.2025.1529804 (PMC11885293; doi:10.3389/fpls.2025.1529804)
Supplement: Supplementary file 4 [file DataSheet1.pdf]

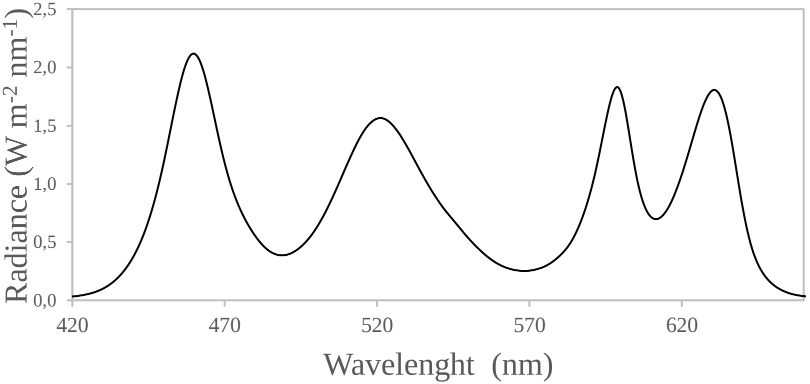

Figure S1. Spectrum of light provided by a multi-channel LEDs lighting system.
The different light treatments were obtained by changing the percentage of the wavelength used. For the blue light treatment, most of the emission (70%) was focused on the blue region (peak at 461 nm), while for the red-light treatment, the 631 wavelength peak radiance was set to be 70% higher than the other channels.
