# Supplementary material for: Blue and red LEDs modulate polyphenol production in Precoce and Tardiva cultivars of Cichorium intybus L
Source: Front Plant Sci. 2025 Feb 21;16:1529804. doi: 10.3389/fpls.2025.1529804 (PMC11885293; doi:10.3389/fpls.2025.1529804)
Supplement: Supplementary file 5 [file Table1.docx]

**Table S1. Polyphenolic composition of *C. intybus* Precoce cultivar after three and four weeks of irradiance with white, blue and red LEDs.** Values are reported as mean of four replicates ± SEM.

| **Precoce** | 3 weeks | | | 4 weeks | | |
| --- | --- | --- | --- | --- | --- | --- |
|  | White LEDs | Blue LEDs | Red LEDs | White LEDs | Blue LEDs | Red LEDs |
| caftaric acid | 0.0358±0.0022 | 0.0455±0.0035 | **-** | 0.0672±0.0069 | 0.0926±0.0032 | **-** |
| chlorogenic acid | 0.1487±0.0165 | 0.2753±0.0172 | **-** | 0.2976±0.0402 | 0.3563±0.0204 | **-** |
| chicoric acid | 0.7343±0.0793 | 1.0750±0.0252 | **-** | 1.0302±0.1406 | 2.2117±0.1633 | **-** |
| quercetin derivative | 0.3642±0.0295 | 1.4659±0.0915 | **-** | 1.6095±0.1565 | 1.9296±0.0567 | **-** |
| quercetin 7-O-glucoside | 0.3797±0.0644 | 0.4897±0.0806 | **-** | 0.5935±0.0756 | 0.9478±0.0685 | **-** |
| epigallocatechin derivative | 0.0650±0.0048 | 0.0888±0.0034 | 0.0956±0.0026 | 0.1287±0.0075 | 0.1737±0.0068 | 0.1504±0.0049 |
| kaempferol-7-O-(6''-O-acetyl)-glucoside | 0.0430±0.0025 | 0.0761±0.0028 | - | 0.0935±0.0017 | 0.1082±0.0045 | - |
| kuromanin | 0.0000±0.0000 | 0.0003±0.0001 | 0.0002±0.0001 | 0.0016±0.0002 | 0.0038±0.0002 | 0.0026±0.0002 |
| cyanidin 3,5 diglucoside | 0.0000±0.0000 | 0.0008±0.0000 | 0.0008±0.0001 | 0.0024±0.0001 | 0.0037±0.0001 | 0.0066±0.0005 |
| cyanidin glucoside 1 | 0.0000±0.0000 | 0.0003±0.0001 | - | 0.0015±0.0001 | 0.0024±0.0001 | - |
| cyanidin 3-O-malonyl glucoside | 0.0000±0.0000 | 0.0056±0.0001 | 0.0024±0.0004 | 0.0162±0.0002 | 0.0517±0.0024 | 0.0163±0.0017 |
| cyanidin glucoside 2 | 0.0000±0.0000 | 0.0004±0.0000 | - | 0.0008±0.0002 | 0.0030±0.0001 | - |
| cichoriin | - | - | 0.0654±0.0045 | - | - | 0.0303±0.0027 |
| 5-O-feruloylquinic acid | - | - | 0.0693±0.0026 | - | - | 0.0209±0.0025 |
| luteolin-7,3'-di-O-glucoside | **-** | **-** | 0.0354±0.0007 | **-** | **-** | 0.0484±0.0023 |
| luteolin 7-glucoside 3'-glucuronide | **-** | **-** | 0.0296±0.0004 | **-** | **-** | 0.0317±0.0032 |
| kaempferol 3-O-glucuronide | **-** | **-** | 2.1133±0.0758 | **-** | **-** | 2.4664±0.0444 |
| kaempferol-3-O-glucosyl-7-O-(6”-O-malonyl)-glucoside | **-** | **-** | 0.1400±0.0038 | **-** | **-** | 0.2255±0.0091 |
| epigallocatechin 3’-O-glucuronide | **-** | **-** | 0.0294±0.0007 | **-** | **-** | 0.0975±0.0015 |
| *Total polyphenols* | 1.7707±0.0474 | 3.5237±0.1695 | 2.5815±0.0858 | 3.8428±0.4206 | 5.8844±0.3115 | 3.0966±0.0449 |
