# Supplementary material for: Blue and red LEDs modulate polyphenol production in Precoce and Tardiva cultivars of Cichorium intybus L
Source: Front Plant Sci. 2025 Feb 21;16:1529804. doi: 10.3389/fpls.2025.1529804 (PMC11885293; doi:10.3389/fpls.2025.1529804)
Supplement: Supplementary file 6 [file Table2.docx]

**Table S2. Polyphenolic composition of *C. intybus* Tardiva cultivar after three and four weeks of irradiance with white, blue and red LEDs.** Values are reported as mean of four replicates ± SEM.

| **Tardiva** | 3 weeks | | | 4 weeks | | |
| --- | --- | --- | --- | --- | --- | --- |
|  | White LEDs | Blue LEDs | Red LEDs | White LEDs | Blue LEDs | Red LEDs |
| caftaric acid | 0.0292±0.0089 | 0.0290±0.0020 | **-** | 0.0261±0.0009 | 0.1316±0.0143 | **-** |
| chlorogenic acid | 0.0485±0.0122 | 0.1354±0.0088 | **-** | 0.0795±0.0097 | 0.3938±0.0238 | **-** |
| chicoric acid | 0.1919±0.0267 | 0.9355±0.0541 | **-** | 0.2752±0.0131 | 2.1900±0.2486 | **-** |
| quercetin derivative | 0.0580±0.0100 | 0.4932±0.0757 | - | 0.1754±0.0146 | 1.1520±0.0951 | **-** |
| quercetin 7-O-glucoside | 0.1341±0.0198 | 0.4739±0.0519 | **-** | 0.2543±0.0421 | 0.8241±0.0681 | **-** |
| quercetin-7-O-(6''-O-malonyl) glucoside | 0.0117±0.0018 | 0.0435±0.0020 | **-** | 0.0318±0.0020 | 0.0574±0.0073 | **-** |
| 3,5-dicaffeoylquinic acid | 0.0222±0.0019 | 0.0392±0.0067 | **-** | 0.0315±0.0010 | 0.0840±0.0130 | **-** |
| isorhamnetin-7-O-glucuronide | 0.0222±0.0019 | 0.0392±0.0133 | **-** | 0.0315±0.0010 | 0.0840±0.0130 | **-** |
| cichoriin | - | - | 0.0458±0.0074 | - | - | 0.1274±0.0044 |
| 5-O-feruloylquinic acid | **-** | **-** | 0.0620±0.0169 | **-** | **-** | 0.6127±0.0184 |
| luteolin-7,3'-di-O-glucoside | **-** | **-** | 0.0164±0.0004 | **-** | **-** | 0.0155±0.0010 |
| luteolin 7-glucoside 3'-glucuronide | **-** | **-** | 0.0026±0.0000 | **-** | **-** | 0.0018±0.0002 |
| kaempferol 3-O-glucuronide | **-** | **-** | 1.6871±0.0396 | **-** | **-** | 0.5785±0.0086 |
| kaempferol-3-O-glucosyl-7-O-(6”-O-malonyl)-glucoside | **-** | **-** | 0.0674±0.0018 | **-** | **-** | 0.0268±0.0014 |
| epigallocatechin derivative | **-** | **-** | 0.1331±0.0034 | **-** | **-** | 0.1343±0.0054 |
| epigallocatechin 3’-O-glucuronide | **-** | **-** | 0.1329±0.0290 | **-** | **-** | 0.0148±0.0004 |
| cyanidin 3 -O-malonyl glucoside | 0.0058±0.0007 | 0.0186±0.0019 | 0.0136±0.0012 | 0.0085±0.0004 | 0.0504±0.0094 | 0.0714±0.0063 |
| *Total polyphenols* | 0.5236±0.0561 | 2.2075±0.1965 | 2.1608±0.0275 | 0.9138±0.0806 | 4.9675±0.3912 | 1.5831±0.0303 |
